# Risk factors for development of nephropathy in patients with a diabetic Charcot foot

**DOI:** 10.1186/s13104-021-05811-5

**Published:** 2021-10-30

**Authors:** Rasmus Bo Jansen, Per E. Holstein, Bo Jørgensen, Klaus Kirketerp Møller, Ole Lander Svendsen

**Affiliations:** 1 Department of Endocrinology, Bispebjerg Hospital, University of Copenhagen, 2400 Copenhagen, NV Denmark; 2 Copenhagen Wound Healing Center, CODIF, Bispebjerg Hospital, University of Copenhagen, 2400 Copenhagen, NV Denmark

**Keywords:** Diabetes mellitus, Charcot foot, Nephropathy, Foot ulcer treatment, Prolonged antibiotics, Risk factor

## Abstract

**Objective:**

Charcot foot is a rare complication to neuropathy and can cause severe foot deformities and ulcerations, which often require prolonged antibiotical treatment. The objective of this retrospective study was to investigate whether this treatment is associated to impaired renal function.

**Results:**

In total, 163 patients were included, of whom 105 (64%) had received β-lactam antibiotics for a mean total duration of 13.0 months. There was a significant increase in the urine albumin/creatinine ratio in the group that received antibiotics (p = 0.017), and the use of antibiotics was associated to a subsequent diagnosis of nephropathy (p = 0.01). Patients treated with antibiotics had a 21.9% risk of developing subsequent nephropathy versus 5.2% for patients not treated with antibiotics. We suggest increased awareness on signs of nephropathy in patients with severe Charcot foot.

**Supplementary Information:**

The online version contains supplementary material available at 10.1186/s13104-021-05811-5.

## Introduction

Charcot foot is a devastating late-stage complication to diabetes. The fractures, joint dislocations and bone remodeling caused by an incorrectly treated Charcot foot precipitate foot deformity, leading to foot ulcerations that are difficult to heal and prone to infections (1–5).

The slow-healing nature of such ulcers commonly means that they require extensive antibiotic treatment. To minimize the risk of infections antibiotic treatment might be initiated without initial identification of any responsible pathogenic species (6–9). Therefore, incorrect offloading of a Charcot foot could necessitate a substantial accumulated antibiotic load for the patient.

One of the most common types of antibiotics used for long-term treatment is oral, semi-synthetic penicillins (i.e., penicillinase-stabilized β-lactam drugs) such as Dicloxacillin or Flucloxacillin. As with most other antimicrobial agents, semi-synthetic penicillins are known to carry a small risk of nephrotoxicity, most notably when used in combination with other drugs (10–12). However, the different classes of β-lactam drugs on the market today are considered safe for standard clinical use, and adverse renal reactions are normally only seen when using high doses in patients with pre-existing impaired renal function (13–15).

Late-stage diabetic complications, such as a Charcot foot, is associated to impaired renal function.

Currently, no data describing adverse renal reactions to long-term antibiotics use in patients with diabetes with a Charcot foot have been published. Thus, the aim of this study was to investigate any association between the use of semi-synthetic penicillins and changes in renal function in patients with diabetes with Charcot foot.

## Main text

### Materials and methods

Data used for this study represent a secondary analysis of a previously described retrospective, longitudinal, observational study on all patients with diabetes treated for a Charcot foot at the Copenhagen Wound Healing Center (CWHC) at Bispebjerg Hospital, Denmark over a 20 year period (16). The patients were identified using ICD-10 codes and followed up through medical records.

It was recorded whether the patients received any long-term treatment with antibiotics from the CWHC (defined as > 1 month's continual treatment). Only prescriptions for antibiotics signed by staff of the CWHC was recorded. Patients were seperated into two groups based upon whether they had received antibiotics for a Charcot-related foot ulcer for more than a month continously during the follow-up period or not.

All available anthropomorphic data were registered (age, sex, diabetes type, duration of diabetes, height, weight and blood pressure). Blood and urine sample values were registered at study inclusion (and up to 6 months prior); as close to introduction of antibiotics treatment as possible (within a margin of 3 months); and when the treatment was stopped (and up to 6 months afterwards).

Hemoglobin A1c was used as a marker for glycemic regulation. Values listed are in IFCC units (mmol/mol), older values measured in the DCCT (%) scale have been converted.

A binary pseudomarker for "Less-than-optimal compliance" was estimated based on the medical records. It was registered as positive if the medical record contained: Repeated missed appointments, incorrect medication intake or insufficient off-loading.

For all occurences of nephropathy in the group it was recorded whether the patient had nephropathy at first contact to the CWHC, or if the patient developed nephropathy during the follow-up period.

Nephropathy was defined using the criteria from the EMPA-REG OUTCOME study as manifest macroalbuminuria (urine albumine/creatinine ratio > 300 mg); progression from microalbuminuria (urine albumine/creatinine ratio between 30 AND 299 mg) to macroalbuminuria; a persistent (two or more consecutive measurements at least a month apart) increase in p-creatinine (above twice the normal range) without another cause present in the records (such as severe infections/septicemia, trauma or hypovolemic shock); initiation of renal dialysis; or death from renal disease (17).

#### Statistics

Data are expressed as [means ± SD] unless otherwise noted. In all tests an α = 0.05 was considered significant. *T*-test and the non-parametric Mann–Whitney rank sum test were used for variance analysis between groups in normally and not normally distributed data sets respectively.

We tested for correlations using the chi-square test (categorical data).

Categorical and continuous data were compared using a logistic regression model. Missing values in binary sets were excluded by row in the relevant analysis, while missing values in multiple point data sets were used as last observation carried forward.

Statistics and generel data handling was done using IBM SPSS Statistics v. 23 by IBM Corporation, SIGMAPLOT v. 11.0.0.77 by Systat Software Inc., Microsoft Excel 2000 v. 9.0.2812 by Microsoft Corporation and Apache OpenOffice 4.0.1 by The Apache Software Foundation.

### Results

Of the 173 patients with diabetes with acute Charcot foot included, data for antibiotics use were available for 163 patients (Table 1).

**Table 1:** Anthropomorphic data at baseline for the patients with diabetes and Charcot foot (n = 163), distributed into a group that received long-term antibiotic treatment for foot infections during their Charcot foot treatment, and a group that did not receive antibiotics for this reason in the period (1996–2015)

	Group with antibiotics (n = 105)	Group without antibiotics (n = 58)	P-value
Age (years)	55.4 ± 10.0	60.1 ± 9.1	0.004^*^
Sex (male/female)^a^	82/23	25/33	0.005^*+^
Diabetes type (I/II)^a^	30/75	10/48	0.109
Duration of diabetes (years)^a^	16.7 ± 12.4	16.6 ± 12.6	0.826
BMI (kg/m^2^)^a^	30.6 ± 9.3	29.1 ± 5.7	0.362
HbA1c (mmol/mol)	71 ± 22	65 ± 19	0.146
Systolic blood pressure (mmHg)	154 ± 22	145 ± 23	0.033^*^
Diastolic blood pressure (mmHg)^a^	83 ± 14	81 ± 15	0.393
P-creatinine (µmol/L)^ab^	93 ± 43	88 ± 29	0.947
U-albumine/creatinine ratio (mg)^ac^	113 ± 257	116 ± 214	0.717

^a^Not normally distributed

^b^Reference level: 60–105 µmol/L for adult males, 45–90 µmol/L for adult females

^c^Reference level: < 30 mg normoalbuminuria, 30–299 mg microalbuminuria, > 300 mg macroalbuminuria

^*^Significant at chosen α-level of 0.05

^+^Significant difference in the distribution of male/female between the two groups

A total of 105 patients (64%) had received long-term antibiotic treatment of infected foot ulcers. Of the remaining 58 patients, some had received antibiotics briefly (cumulatively < 1 month during follow-up).

The group that received antibiotics were significantly younger (*t*-test, p = 0.004), had a higher ratio of males (rank sum test, p = 0.005) and had significantly higher systolic blood pressure (*t*-test, p = 0.033) than the group that did not receive antibiotics.

The main oral antibiotic used was the semi-synthetic β-lactam drug Dicloxacillin, which was used for a mean total duration of 13.0 months (median 9.0 months; range 1–45 months). The primary dosage regimes were Dicloxacillin 1 g 3 times/day or 1 g 4 times/day in case of obese patients.

There was a significant increase in the u-albumine/creatinine ratio in the group that received antibiotics from 113 to 431 mg (rank sum test, p = 0.017), as well as in the p-creatinine levels from 93 to 154 µmol/L (rank sum test, p = 0.037). There were no differences in the group without antibiotics in either the u-albumine/creatinine ratio 116 to 194 mg (rank sum test, p = 0.440) or p-creatinine 88–97 µmol/L (rank sum test, p = 0.501).

A total of 51 patients were diagnosed with nephropathy either before inclusion (n = 25), or during follow-up (after diagnosis of Charcot foot) (n = 26).

A contingency table of the development of nephropathy and use of antibiotics is listed as Table 2. It shows that 23 of the 26 patients who developed nephropathy during follow-up had received long-term antibiotics for a foot related infection. Of the patients who did not develop nephropathy (137 out of the 163) 82 had received long-term antibiotics while 55 had not. This equals a relative risk of 4.2 between development of nephropathy and use of long-term antibiotics.

**Table 2:** Long-term use of beta-lactam antibiotics for foot infections and development of nephropathy in patients with diabetes with Charcot foot

	Develops nephropathy after onset of Charcot foot		n
	Yes (%)	No (%)	
Antibiotics (for foot related issues)			
Yes	23 (21.9%)	82 (78.1%)	105
No	3 (5.2%)	55 (94.8%)	58
n	26 (16%)	137 (84%)	163

In a chi-square test, the use of antibiotics for foot infections was associated with a subsequent diagnosis of nephropathy (p = 0.01).

Using a logistical regression, the duration of antibiotics use was related to the subsequent diagnosis of nephropathy with increasing dose significantly increasing the risk of nephropathy (p = 0.005; Hosmer–Lemeshow Statistic: 9.548 (p = 0.298), R_L_^2^ = 0.064) (Additional File 1: Figure S1).

Less-than-optimal compliance and HbA1c were used as pseudomarkers to approximate negligent self-care and blood glucose regulation respectively, and were correlated to the length of antibiotics use using Spearman's Rank order tests. Both were positively correlated, however with weak correlation coefficients (length of antibiotics use and HbA1c; p = 0.006, r = 0.294)(length of antibiotics use and less-than-optimal complicance; p < 0.001, r = 0.304).

A combined logistic regression analysis model was performed to approximate the individual impact of each of the following factors: baseline p-creatinine, baseline u-albumine/creatinine ratio, length of antibiotics use, HbA1c and less-than-optimal compliance. Only baseline p-creatinine and length of antibiotics use remained significant contributing factors to the development of nephropathy (p = 0.043 and p = 0.048 respectively) (Additional File 2: Figure S2).

### Discussion

We have performed a retrospective study of the use of long-term oral antibiotics for foot infections in patients with diabetes and Charcot foot.

We found an association between the use of Dicloxacillin and worsening renal function, with long-term use being associated with a fourfold increase in the risk of developing diabetic nephropathy. Furthermore, we found that increased duration of Dicloxacillin treatment was associated with increased risk of developing nephropathy. As expected, we found that elevated baseline p-creatinine levels were also associated with increased risk of developing nephropathy.

The association between diabetic foot ulcers and kidney damage is well-known (18–21). It might, in part, be caused by extended inflammatory conditions as well as delayed clearance of certain neurotoxic waste products (22–24). Furthermore, nephropathy is associated to major complications in patients with a Charcot foot (such as ulcerations, deformities or amputation) (25). However, the chronological causality of this is not clear.

There is little-to-no evidence suggesting that extended usage of large doses of Dicloxacillin might be nephrotoxic in an unselected population. The few papers available are of older date and/or concern short-term use as prophylaxis before surgery (26–31). The apparant nephrotoxicity of Dicloxacillin in these cases could very well be due to usage in patients with a renal vulnerability consisting of old age and large orthopedic surgery.

It is unknown whether some dysregulated patients with diabetes have a similiar renal vulnerability. Our data could well express such a renal vulnerability in the subgroup of the patients who were at higher risk due to other factors (such as extended hyperglycaemia or reduced compliace to treatment).

Furthermore, it should be remembered that this class of antibiotics is important for treatment of foot infections, and can be vital for limb salvage.

In conclusion, in this cohort, long-term treatment with the penicillinase-stabilized β-lactam antibiotic Dicloxacillin for foot related infections, in patients with diabetes and a Charcot foot, was associated with a fourfold increase in risk of developing nephropathy. We propose that a need for long-term antibiotics might be seen as a marker for Charcot patients in high risk of developing renal impairment.

### Limitations

The main limitations of this study are that it is retrospective, observational and with data drawn from standard medical records. Hence we are not able to draw any conclusions on causality.

Data show that the patients prescribed antibiotics also had the most advanced diabetes and thus a higher risk of end stage complications (including nephropathy) to begin with. Most importantly for the development of renal impairment, they also had a significantly higher systolic blood pressure. In addition, they had worse blood glucose regulation and worse estimated compliance.

Whether these factors can attest for the entirety of the difference between the two groups we cannot say. It could be speculated that the need for long-term antibiotics for foot ulcers in a Charcot patient is in fact a marker for a complicated patient with a more-than-average risk of end-stage complications.

## Supplementary Information

**Additional file 1: Figure S1.** Cumulated maximum length of antibiotics use for Charcot-related foot ulcers and subsequent development of nephropathy (n = 91).

**Additional file 2: Figure S2.** Multiple Logistic Regression.

## Data Availability

All data and material used will be provided, in anonymized form, upon request to the corresponding author (RBJ) at rasmus.bo.jansen@regionh.dk.

## Abbreviations

CWHC: Copenhagen Wound Healing Center
ICD-10: International classification of diseases, version 10
IFCC: International Federation of Clinical Chemistry
DCCT: Diabetes control and complications trial
SD: Standard deviation
HbA1c: Haemoglobin A1c
