# Supplementary figures and images for: Risk factors for development of nephropathy in patients with a diabetic Charcot foot
Source: BMC Res Notes. 2021 Oct 30;14:403. doi: 10.1186/s13104-021-05811-5 (PMC8557477; doi:10.1186/s13104-021-05811-5)

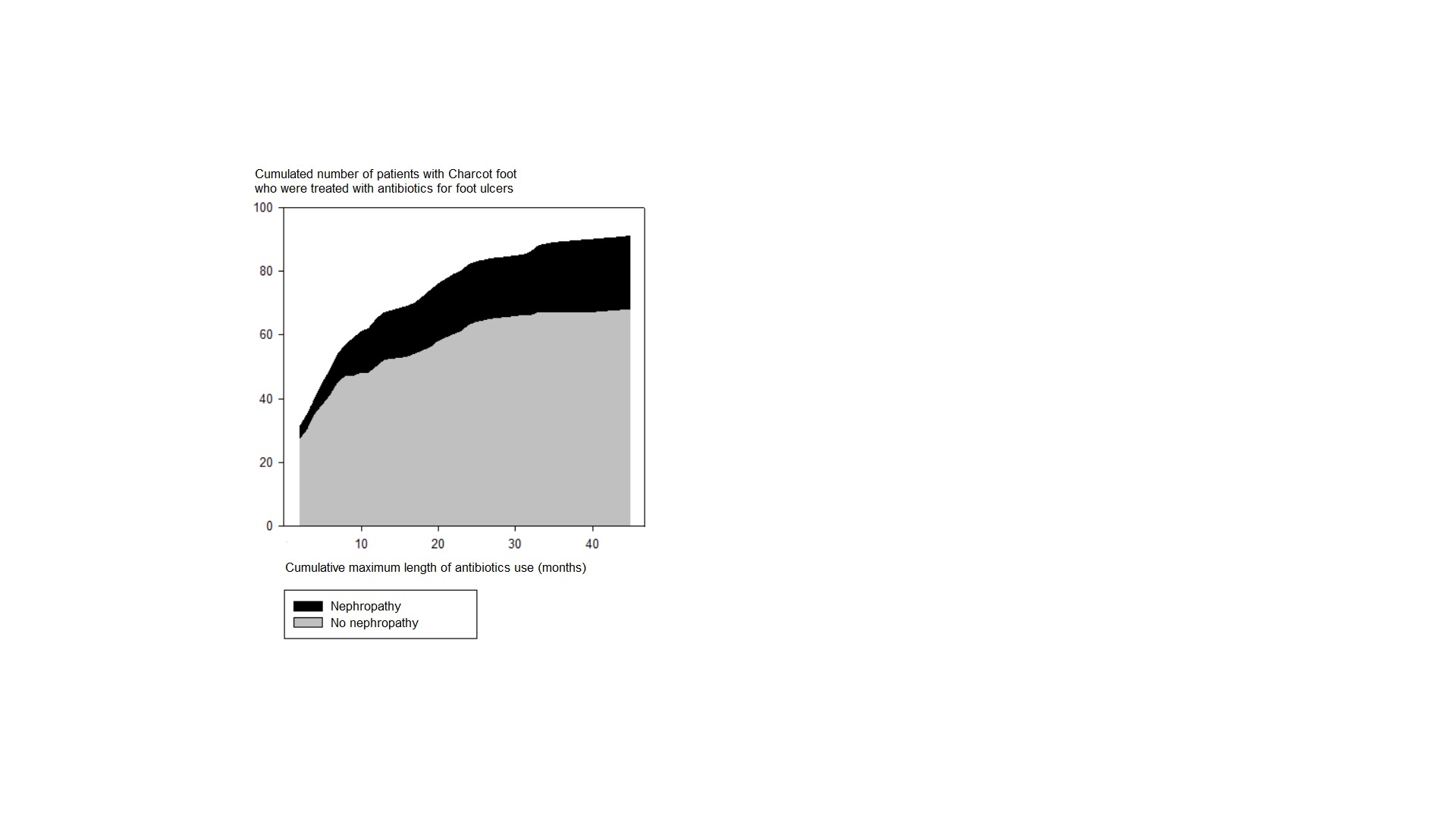

Supplement: Supplementary file 1 — Additional file 1: Figure S1. Cumulated maximum length of antibiotics use for Charcot-related foot ulcers and subsequent development of nephropathy (n = 91). [file 13104_2021_5811_MOESM1_ESM.jpg]
