# Supplementary material for: Risk factors for development of nephropathy in patients with a diabetic Charcot foot
Source: BMC Res Notes. 2021 Oct 30;14:403. doi: 10.1186/s13104-021-05811-5 (PMC8557477; doi:10.1186/s13104-021-05811-5)
Supplement: Supplementary file 2 — Additional file 2: Figure S2. Multiple Logistic Regression. [file 13104_2021_5811_MOESM2_ESM.doc]

**Additional file 2: Figure S2.**

**Multiple Logistic Regression**

Logit P = -8.091 + (0.0899 * A) + (0.494 * B) - (0.000974 * C) + (0.0366 * D) + (0.0173 * E)

A = Total duration of antibiotics

B = Less-than-optimal compliance

C = Baseline urine albumin/creatinine ratio

D = Baseline creatinine

E = HbA1c

**Pearson Chi-square Statistic:** 36.134 (P = 0.415)

**Likelihood Ratio Test Statistic:** 17.315 (P = 0.004)

**-2*Log(Likelihood) =** 20.532

**Hosmer-Lemeshow Statistic:**  6.914 (P = 0.546)

**Ind. Variable Coefficient P value**

Constant -8.091 0.011

A 0.0899 0.043

B 0.494 0.699

C -0.000974 0.733

D 0.0366 0.048

E 0.0173 0.521
